# Learning about Expectation Violation from Prediction Error Paradigms – A Meta-Analysis on Brain Processes Following a Prediction Error

**DOI:** 10.3389/fpsyg.2017.01253

**Published:** 2017-07-28

**Authors:** Lisa D’Astolfo, Winfried Rief

**Affiliations:** Department of Clinical Psychology and Psychotherapy, Philipps University of Marburg Marburg, Germany

**Keywords:** expectation violation, prediction error, fMRI, meta-analysis, striatum, insula

## Abstract

Modifying patients’ expectations by exposing them to expectation violation situations (thus maximizing the difference between the expected and the actual situational outcome) is proposed to be a crucial mechanism for therapeutic success for a variety of different mental disorders. However, clinical observations suggest that patients often maintain their expectations regardless of experiences contradicting their expectations. It remains unclear which information processing mechanisms lead to modification or persistence of patients’ expectations. Insight in the processing could be provided by Neuroimaging studies investigating prediction error (PE, i.e., neuronal reactions to non-expected stimuli). Two methods are often used to investigate the PE: (1) paradigms, in which participants passively observe PEs (”passive” paradigms) and (2) paradigms, which encourage a behavioral adaptation following a PE (“active” paradigms). These paradigms are similar to the methods used to induce expectation violations in clinical settings: (1) the confrontation with an expectation violation situation and (2) an enhanced confrontation in which the patient actively challenges his expectation. We used this similarity to gain insight in the different neuronal processing of the two PE paradigms. We performed a meta-analysis contrasting neuronal activity of PE paradigms encouraging a behavioral adaptation following a PE and paradigms enforcing passiveness following a PE. We found more neuronal activity in the striatum, the insula and the fusiform gyrus in studies encouraging behavioral adaptation following a PE. Due to the involvement of reward assessment and avoidance learning associated with the striatum and the insula we propose that the deliberate execution of action alternatives following a PE is associated with the integration of new information into previously existing expectations, therefore leading to an expectation change. While further research is needed to directly assess expectations of participants, this study provides new insights into the information processing mechanisms following an expectation violation.

## Introduction

Patients’ expectations have a great influence on their treatment and outcomes in psychotherapy (Greenberg et al., 2006), medical conditions as well as in patients undergoing surgery (Auer et al., 2016; Rief and Glombiewski, 2016). In addition, negative expectations about psychological interventions may lead to negative effects of psychotherapy (Ladwig et al., 2014). Rief et al. (2015) have proposed to consider dysfunctional expectations to be core features of mental disorders. It has been argued that dysfunctional behavior is guided by dysfunctional expectations of situational associations and outcomes. Hence, behavioral therapy would only be successful if there is a change of the dysfunctional expectations guiding the behavior. These dysfunctional expectations are pre-existing assumptions about contingencies with a high subjective associative strength, i.e., subjective certainty. Patients would have to experience an expectation violation, i.e., a state, in which the expected outcome and the actual outcome differ, to induce a change in their expectations about the contingencies. This corresponds to a relearning of the contingencies, i.e., a state, in which they perceive a difference between expected outcome and the actual outcome, which would induce a change in their expectations about the contingencies. It is hypothesized that depending on various information processing variables, expectations might either be changed or maintained after an expectation violation situation. Thus, the relearning is either successful and persists over time or the relearning might be only temporary or depending on contextual factors.

The particular mechanisms underlying the information processing and the persistence and change of expectations have remained unclear. Clinical observations suggests that patients with mental disorders are particularly resistant to expectation change and the perception on expectation violations (Rief et al., 2015; Rief and Glombiewski, 2016). There are promising new approaches examining immunization as one of the processing strategies following expectation violation (Kube et al., 2016). This could explain why even after a successful expectation violation, the expectation is not changed. The patients perceive the violation of their pre-existing expectation but attribute the situation to contextual factors, e.g., the setting. Thus, the confrontation with an aversive stimulus with aim of reducing an emotional response, as is commonly used in psychotherapeutic settings, might not always be enough to induce a persistent expectation change. Craske et al. (2014) proposed methods of maximizing such exposure techniques, which are supposed to increase the inhibitory learning of the old expectation about the contingencies. One of these methods is the active testing of the pre-existing expectation. This is suggested to facilitate the relearning of the contingencies and to stabilize the newly learned expectation, thus inducing an expectation change.

The change of dysfunctional expectations is theorized as a crucial mechanism for therapeutic success. The investigation of cognitive processes facilitating an expectation change vs. maintenance following an expectation violation might pose a promising approach for cognitive behavioral therapy. Thus, we propose to compare the cognitive processing of a more passive confrontation with the aim of reducing an emotional response and an active approach by testing the expectation.

The neuroimaging research on learning provides experimentally designed expectation violations. One of the concepts consistently associated with successful learning is the so-called prediction error, i.e., the neurological response to an unexpected stimulus. Learning research has mainly focused on reinforcement learning, whereby the expectations comprises predictions about reward and/or punishment (Karuza et al., 2014). Many studies use partial reinforcement or probabilistic learning paradigms. It can be argued that changes in behavioral strategies in these paradigms also reflect changes in underlying expectations regarding the contingencies of reward and punishment. Hence, in paradigms, in which no behavioral adaptation is necessary, i.e., a passive observation of contingencies, might diminish the attention on expectation violations. We argue that participants in both paradigms compute prediction errors and their relearning of the contingencies is successful. In alignment with the approach by Craske et al. (2014) to maximize inhibitory learning by actively testing the expectation, we hypothesize a different cognitive processing of “active” paradigms, which encourage a behavioral adaptation and “passive” paradigms, in which contingencies are observed. Since the concepts of prediction error and expectation violation are identical in matters of meaning for the preexisting expectation, it seems likely that clinical research can benefit from an insight of neuroimaging research on prediction error. Examining the functional magnetic resonance imaging (fMRI) results provided by research on prediction error might provide insights in the cognitive processes associated with the information processing during expectation violations.

Our aim is to review fMRI studies investigating two different prediction error paradigms. The first paradigm encourages strategic behavioral changes throughout the course of experiments while the second one requires a passive observation. A contrast analysis will be performed to identify differences in brain activity between these two paradigm categories. A meta-analysis summarized the current findings on brain areas associated with prediction error (Garrison et al., 2013). They found a consistent association of the pallidum, the striatum and medio-frontal structures with prediction error. These structures are also associated with the fronto-striatal circuits. The circuit is defined as circular connections between the caudate nucleus, putamen, thalamus and prefrontal regions (Leh et al., 2007). Dysfunctions in this circuit are associated with impaired behavioral adaptation such as poor set shifting performance, e.g., in a go/no-go tests or stimulus-bound behavior (Mega and Cummings, 1994). Several disorders are linked to fronto-striatal circuit dysfunctions, such as Huntington’s disease (Beste et al., 2012), Parkinson’s Disease (Owen, 2004) and obsessive-compulsive disorder (Maltby et al., 2005; Marsh et al., 2014). All clinical pictures are associated with behavioral and cognitive perseverations (Mega and Cummings, 1994). It therefore seems likely to assume the fronto-striatal circuit to be involved in the expectation violation processing and the resulting expectation and behavioral adaptation. We will perform a meta-analysis involving prediction error followed by a behavioral adaptation to an uncertain environment. We expect a consistent activation in the striatum and media-frontal areas.

## Materials and Methods

### Literature Selection

**FIGURE 1:**
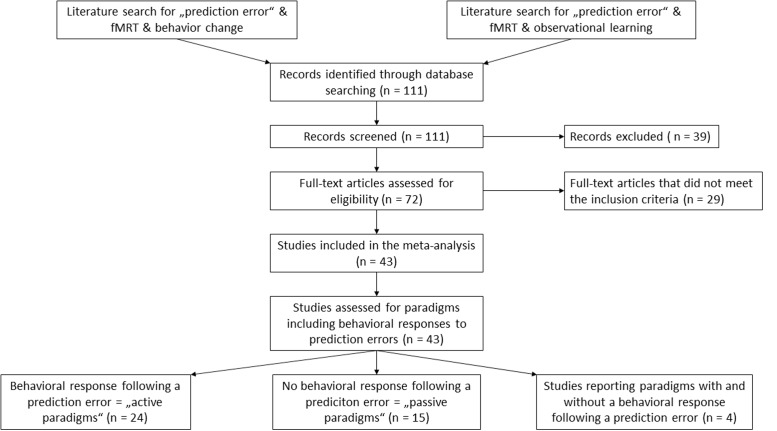
Overview of the literature selection process.

We conducted a systematic literature search to identify neuroimaging studies of prediction error using PubMed^1^, Web of Science^2^, and Neurosynth^3^ databases. We searched for articles in the English language using the keywords “prediction error” AND “fMRI” and did not specify a time span for date of publication. The search revealed 8’610 results as of July 2016. To narrow the results, a second search was performed using the keywords “prediction error” AND “fMRI” AND “behavior change” as well as “prediction error” AND “fMRI” AND “observational learning”. Again, no time span was specified. These searches revealed 111 results and four results, respectively. The abstracts of these articles were examined to select potential matches for our inclusion criteria. We also scanned the reference lists of the results to search for additional articles, which met our inclusion criteria. We retrieved the full text of 72 articles for further examination. We predefined study selection criteria to minimize ambiguousness in the study selection. The criteria can be requested of the corresponding author. Studies were included when they met the following criteria: (1) experimental prediction error paradigm and (2) report of voxel-wise-brain analysis for a prediction error main effect, which yielded a total of 59 articles. We excluded studies which did not report prediction error for healthy adults or used medication in their experiment (*n* = 6 studies excluded). We did this to include only prediction errors which arise from an unexpected change in contingencies in alignment with the clinical model. Of these studies, we precluded those articles failing to experimentally induce a prediction error by changing the contingencies between stimuli and outcome (*n* = 10 studies excluded). A flowchart of the selection process is shown in **Figure 1**. The studies included in the meta-analysis are listed in **Table 1**.

**Table 1:** Overview of the prediction error studies included in the meta-analysis.

Reference	Number of subjects	Task	Behavioral adaptation possible?
Abler et al., 2006	11	Reward probability	Yes
Behrens et al., 2007	18	Reward probability	Yes
Cohen, 2007	17	Reward probability	Yes and No
Daniel and Pollmann, 2012	18	Visual classification	Yes and No
Delgado et al., 2008	11	Aversive conditioning	No
den Ouden et al., 2010	20	Auditory classification	Yes
Diuk et al., 2013	28	Reward probability	Yes
Gläscher et al., 2010	18	Markov decision task	Yes
Gläscher et al., 2009	20	Reversal learning	Yes
Gradin et al., 2011	20	Reward learning	Yes
Ham et al., 2013	35	Simon Task	Yes
Hampton et al., 2006	16	Reversal learning	Yes
Haruno and Kawato, 2006	20	Association learning	Yes
Hester et al., 2010	16	Association learning	Yes
Hester et al., 2008	17	Spatial learning task	Yes and No
Kahnt et al., 2011	20	Orientation discrimination	Yes
Kim et al., 2006	16	Instrumental choice	Yes
Klein et al., 2007	26	Probabilistic learning	Yes
Kumar et al., 2008	18	Reward conditioning	No
Landmann et al., 2007	16	Trial-and-error learning	Yes
Li et al., 2011	17	Fear conditioning	No
Li et al., 2006	46	Reward probability	Yes
Limongi et al., 2013	15	Michotte’s Launching effect	No
McClure et al., 2003	18	Reward conditioning	No
Metereau and Dreher, 2013	20	Pavlovian conditioning	No
Morris et al., 2012	16	Reward conditioning	Yes
Murray et al., 2007	12	Reward learning	Yes
Niv et al., 2012	16	Reward probability	Yes
O’Doherty et al., 2004	12	Reward conditioning	Yes
Ploghaus et al., 2000	12	Fear conditioning	No
Ramnani et al., 2004	6	Instrumental conditioning	No
Rodriguez et al., 2006	15	Visual classification	Yes
Schiller et al., 2008	17	Fear conditioning	No
Schlerf et al., 2012	10	Motoric learning	No
Schonberg et al., 2010	17	Reward probability	Yes
Seymour et al., 2007	24	Reward conditioning	No
Seymour et al., 2005	19	Aversive conditioning	No
Spoormaker et al., 2011	40	Fear conditioning	No
Takemura et al., 2011	23	Reward conditioning	No
Tobler et al., 2006	22	Reward blocking	Yes and No
Valentin and O’Doherty, 2009	17	Reward probability	Yes and No
Watanabe et al., 2013	20	Reward probability	Yes


### Contrast Selection

We included all analyses which contrasted prediction error brain activity with brain activity during expectation confirming trails or paradigm specific variations of these contrasts. Of the 43 studied that met all inclusion criteria, we included 60 contrasts in the analysis. If the coordinates were reported in Talairach space they were transformed to Montreal Neurologic Institute (MNI) space using the GingerAle software (Eickhoff et al., 2009, 2012), which utilizes the icbm2tal transform algorithm (Lancaster et al., 2007). In total, we included 446 foci into the analysis.

### Activation Likelihood Estimation (ALE)

We performed an activation likelihood estimation (ALE) analysis using the Software GingerAle (Eickhoff et al., 2009, 2012). The algorithm assesses above-chance clustering between experiments, using a probability distribution centered at each of the foci used in the analysis. Since the spatial relationship is assumed to be fixed in each experiment, the ALE analysis infers random effects (Eickhoff et al., 2009). We used the algorithm described in Turkeltaub et al. (2012), which organizes the foci by subject group (as opposed to study affiliation). This prevents an influence of multiple foci from one experiment on the Meta-Analysis results (Turkeltaub et al., 2012). We performed three Meta-Analyses: (1) studies which encourage a behavioral strategic adaptation following a prediction error, (2) studies, which employed a passive observational paradigm, and (3) an analysis of all studies, which was necessary to perform the contrast analysis. In line with previous studies (Garrison et al., 2013), we defined a false discovery rate (FDR) method with *p* < 0.05 and a minimal cluster volume of 50 mm^3^. We then performed a contrast analysis of the “active” behavioral subset and the “passive” observational study subset. This analysis allows the subtraction of two datasets to compare differences in brain activity between these two. To this end, a pooled dataset is created, which then serves as basis for two randomly created datasets with the same number of foci as the original datasets. A permutation of subtractions of simulated datasets are compared to the results of the original datasets. We used an uncorrected *p*-value *p* < 0.05 since the single analyses were already corrected with FDR (Eickhoff et al., 2011). We chose a minimal cluster volume of 50 mm^3^ for the contrast analysis. Papaya^4^ was used to superimpose the ALE cluster results on a T1 brain template (Colin27_T1_seg_MNI.nii^5^).

## Results

### Meta-Analysis across All Studies of Prediction Error

**FIGURE 2:**
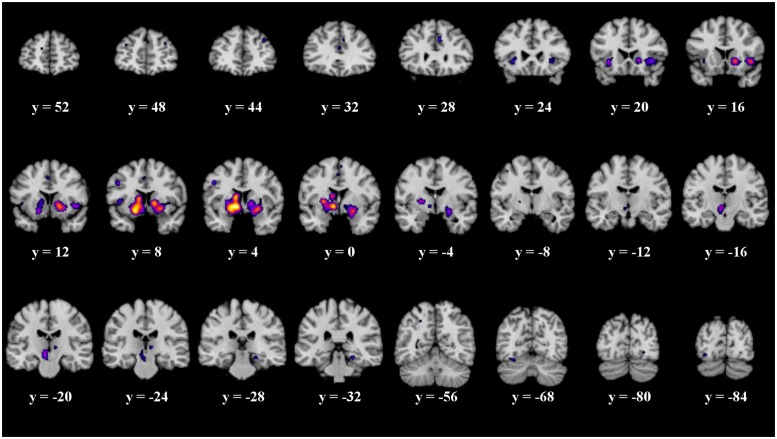
Overview of the meta-analysis results for all studies (*p* < 0.05, FDR). The significant clusters are comprised of activity in the superior frontal gyrus, middle frontal gyrus, anterior cingulate, cingulate gyrus, claustrum, insula, caudate head, precentral gyrus, putamen, lateral globus pallidus, caudate body, red nucleus, thalamus, parahippocampal gyrus, superior parietal lobule, declive, lingual gyrus and the fusiform gyrus (from left to right). MNI coordinates are presented below each coronal view.

Twenty-one significant clusters were identified by the ALE meta-analysis of all 43 studies. The results show activation in the right basal ganglia and the right insula (see details in **Figure 2** and **Table 2**). There was no clear indication of laterality in the main analysis.

**Table 2:** Details of the clusters revealed by the analysis across all studies.

Cluster		MNI coordinates			Cluster size [mm^3^]
		*X*	*Y*	*Z*	
Caudate Head	R	12	8	0	10’712
	R	16	14	-4	
Putamen	R	24	6	-8	
	R	28	4	6	
Insula	R	42	16	-4	
Lateral Globus Pallidus	R	22	0	-12	
Claustrum	R	30	22	-4	
Medial Globus Pallidus	L	-10	2	-4	9’296
Lateral Globus Pallidus	L	-16	6	-6	
Putamen	L	-18	6	-10	
	L	-26	0	4	
Caudate Body	L	-12	6	8	
Red Nucleus	L	-8	-18	-6	1’704
Claustrum	L	-32	22	-6	928
Precentral Gyrus	L	-44	8	4	528
	L	-46	6	34	488
Cingulate Gyrus	R	6	28	32	368
Parahippocampal Gyrus	R	22	-30	-14	312
Middle Frontal Gyrus	R	34	44	30	312
Superior Frontal Gyrus	R	32	48	24	
Declive	L	-30	-68	-18	272
Fusiform Gyrus	L	-34	-86	-10	232
Cingulate Gyrus	L	-4	10	42	232
Thalamus	R	8	-22	2	224
Anterior Cingulate	L	0	34	18	216
Cingulate Gyrus	L	-2	0	48	120
Superior Frontal Gyrus	L	-12	54	18	104
Lingual Gyrus	R	24	-82	-6	88
Precentral Gyrus	L	-40	-10	54	72
Medial Frontal Gyrus	R	4	0	60	64
Middle Frontal Gyrus	L	-32	48	24	56
Superior Parietal Lobule	L	-26	-56	44	56


*Threshold Method = FDR; Thresholding Value = 0.05; Chosen min. cluster size = 50 mm^3^; R, Right; L, Left*.

### Meta-Analyses for Behavioral and Observational Paradigms

**FIGURE 3:**
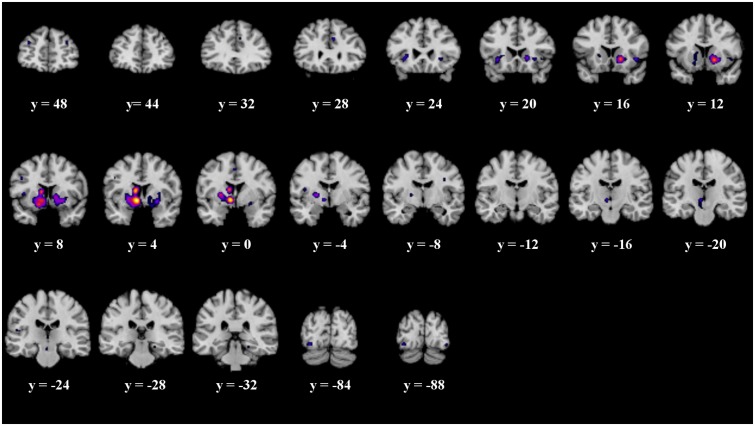
Overview of the meta-analysis results for the studies reporting active behavioral paradigms (*p* < 0.05, FDR). The significant clusters are comprised of activity in the superior frontal gyrus, middle frontal gyrus, anterior cingulate, cingulate gyrus, claustrum, insula, caudate head, inferior frontal gyrus, caudate body, putamen, medial globus pallidus, thalamus, substancia nigra, red nucleus, transverse temporal gyrus, parahippocampal gyrus, fusiform gyrus and inferior occipital gyrus (from left to right). MNI coordinates are presented below each coronal view.

When analyzing all prediction error studies, which employed a behavioral reaction following a prediction error, the ALE meta-analysis revealed 17 significant clusters. We found activation in the striatum, the insula and the claustrum (see details in **Figure 3** and **Table 3**).

**Table 3:** Details of the clusters revealed by the analyses of the behavioral and passive studies.

Cluster		MNI coordinates			Cluster size [mm^3^]
		*X*	*Y*	*Z*	
**Behavioral paradigms**					
Medial Globus Pallidus	L	-10	2	-4	8’528
Caudate Body	L	-10	4	12	
Putamen	L	-16	0	4	
	L	-20	16	2	
Claustrum	L	-32	22	-4	
Caudate Head	R	16	14	-4	4’896
Claustrum	R	30	22	-4	
Putamen	R	28	4	6	
Thalamus	L	8	-18	-4	608
Substantia Nigra	L	-8	-20	-14	
Red Nucleus	L	-4	-22	-18	
Insula	R	42	16	-4	480
Fusiform Gyrus	L	-34	-86	-10	344
Cingulate Gyrus	R	6	28	30	208
Insula	L	-32	48	24	144
Inferior Frontal Gyrus	L	-46	6	32	136
Insula	L	-42	-4	14	120
Parahippocampal Gyrus	R	22	-30	-14	104
Cingulate Gyrus	L	-2	0	48	104
Superior Frontal Gyrus	R	32	48	24	96
Inferior Occipital Gyrus	R	38	-88	-12	88
Transverse temporal Gyrus	L	-52	-24	12	64
Anterior Cingulate	L	-2	34	16	64
Middle Frontal Gyrus	R	36	46	32	56
**Passive paradigms**					
Putamen	L	-20	6	10	720
Lateral Globus Pallidus	R	20	-2	-12	680
Putamen	R	22	6	-10	
Declive	L	-30	-68	-18	80
Lingual Gyrus	R	24	-82	-6	80


*Threshold Method = FDR; Thresholding Value = 0.05; Chosen min. cluster size = 50 mm^3^; R, Right; L, Left*.

**FIGURE 4:**
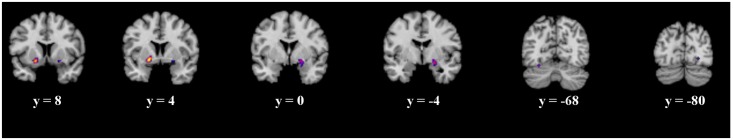
Overview of the meta-analysis results for the studies reporting passive observational paradigms (*p* < 0.05, FDR). The significant clusters are comprised of activity in the putamen, lateral globus pallidus, declive and the lingual gyrus (from left to right). MNI coordinates are presented below each coronal view.

The ALE meta-analysis of all prediction error studies, which employed a passive paradigm revealed four significant clusters. We found activation in the putamen, the lateral globus pallidus, declive and the lingual gyrus (see details in **Figure 4** and **Table 3**).

In both analyses, no clear indication of laterality was found.

#### Subtraction Analysis

**FIGURE 5:**
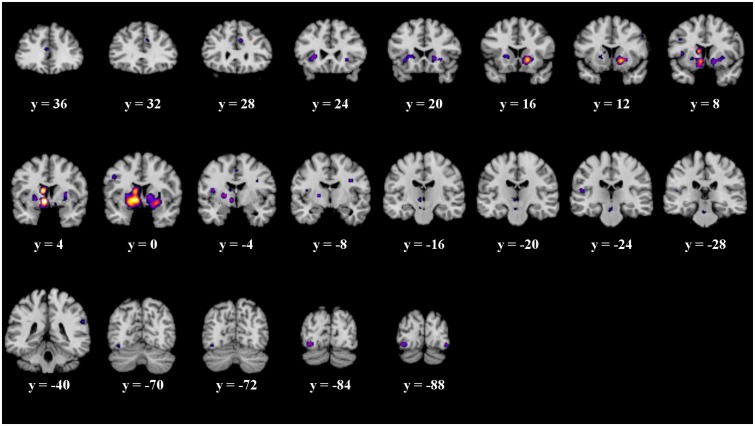
Overview of the meta-analysis results of the behavior – no behavior subtraction analysis (*p* < 0.05, uncorrected). The significant clusters are comprised of activity in the putamen, caudate head, caudate body, insula, medial globus pallidus, and the fusiform gyrus (from left to right). MNI coordinates are presented below each coronal view.

The details of the ALE subtraction analysis are shown in **Table 4** and **Figure 5**. In the contrast behavior – passive, we found five significant clusters, comprising parts of the striatum, the insula and the fusiform gyrus. There was a tendency of left sided structures to be more active in prediction error paradigms encouraging behavioral adaptation. We found no significant clusters in the contrast passive – behavior.

**Table 4:** Details of the clusters revealed by subtraction analysis.

Cluster		MNI coordinates			Cluster size [mm^3^]
		*X*	*Y*	*Z*	
**Behavior – no behavior**					
Medial Globus Pallidus	L	-8	2	-12	1’296
Caudate Body	L	-6	2	18	848
	L	-12	4	18	
	L	-10	8	16	
	L	-10	2	14	
	L	-6	4	12	
	L	-14	-2	16	
Caudate Head	R	12	12	-8	704
Putamen	R	20	16	-4	
	R	24	16	-2	
Fusiform Gyrus	L	-38	-84	-12	200
	L	-34	-84	-8	
Insula	L	-42	-6	16	72
**No behavior – behavior**					
**n.s.**					


*Threshold Method = Uncorrected P-value; Thresholding Value = 0.05; Thresholding Permutations = 10000; Chosen min. cluster size = 50 mm^3^; R, Right; L, Left*.

It is often suggested to apply corrected thresholds to the contrast analyses, such as a FDR threshold. Therefore, we replicated the subtraction analyses with more conservative thresholds. We applied a corrected FDR threshold of *p* < 0.05 to the subtraction analysis. We found no significant clusters in the contrast passive – behavior. The significant clusters of the contrast behavior – passive do not survive the corrected threshold.

## Discussion

We performed a subtraction analysis of two different prediction error paradigms. One encourages a behavioral adaptation to changing contingencies while the second paradigm requires a passive observation of contingencies. Our aim was to gain a better understanding of why and how psychological interventions focusing on expectation violation lead to behavioral changes in some but not all cases. Therefore, we analyzed differences in prediction error involving on one hand the execution of an action alternative and on the other hand no behavioral change. We wanted to identify cognitive processes being involved in underlying expectations about contingencies guiding the behavior. As a major result when contrasting studies employing the two paradigms discussed earlier, we found significantly more activation in the left medial globus pallidus, the left caudate body, the right caudate head and putamen as well as the left fusiform gyrus and the left insula.

### All Studies of Prediction Error

When performing a meta-analysis containing all prediction error studies our results are in line with previous research (Garrison et al., 2013). We found activation in the striatum, the insula, thalamus as well as fronto-medial structures. The Putamen and the Caudate body are part of the striatum whose association with memory processes is consistent with previous literature (Grahn et al., 2009; Provost et al., 2015). The insula has been associated primarily with fear conditioning (Kircher et al., 2013) but also with reinforcement learning for reward (Lawrence et al., 2014) as well for avoidance learning (Palminteri et al., 2012). Consistent with our hypothesis we also found activation in the areas associated with the fronto-striatal circuits (Leh et al., 2007). In addition to the striatum, we found activation in the globus pallidus, the thalamus and frontal structures, i.e., the left superior, media and middle frontal gyrus.

### Prediction Error Followed by Behavioral Adaptation or Passive Observation

When contrasting the differences in neuronal activity of prediction errors computed in active behavioral adaptation and passive observational paradigms we found higher activation in the striatum, the insula and the fusiform gyrus.

The medial globus pallidus is part of the four corticostriatal loops, which are responsible for executive function, visual processing, motor function and motivational evaluation (Seger, 2006). It serves as an output nucleus of the basal ganglia and projects to the thalamus, the centromedian nucleus, and the pedunculopontine nucleus (Nauta and Mehler, 1966). These structures are associated with goal-directed motor actions as well as reward learning and evaluation (Hong and Hikosaka, 2008; Haber and Knutson, 2009; Sescousse et al., 2013).

The putamen is associated with novel motoric executions as well as in ambiguous action tendencies, i.e., if the best motoric strategy is unclear (Grahn et al., 2009). Moreover, due to findings of strong connectivity of the putamen with prefrontal regions, it is suggested that the putamen has a cognitive rather than solely motoric function (Provost et al., 2015).

The caudate body has been shown to be involved in cognitive tasks such as categorization and reward information assessment in monkeys (Yanike and Ferrera, 2014) as well as in humans (Packard and Knowlton, 2002). Further, it has been suggested, that the caudate nucleus is involved in evaluating outcomes post-decision (Badre, 2012; Kepecs and Mainen, 2012).

Most studies do not specifically differentiate between distinct parts of the striatum, but investigate the striatum in its entirety. The striatum has been associated with strategizing in avoidance learning (Palminteri et al., 2012), failure or success to learn associations in instrumental conditioning (Schönberg et al., 2007; Horga et al., 2015), decision making and motor initiation (Nagano-Saito et al., 2014).

The insula is associated with the perception and processing of interoception of emotional states (Zaki et al., 2012; Simmons et al., 2013).

The fusiform gyrus is associated with facial and body recognition (Peelen and Downing, 2005) as well as a sensitivity to visual words (McCandliss et al., 2003). The area of the fusiform gyrus showing peak activation is also associated with object recognition (Bar et al., 2001).

The functions of these areas can be incorporated into the processing of prediction errors computed in a behavioral paradigm. The higher activation of the putamen in prediction errors with behavioral changes might be due to the determination of a novel motoric behavior and its initiation. Due to its’ evaluative properties, the caudate nucleus could function as a constant evaluation unit, comparing expected and actual outcomes. The involvement of the insula cannot be explained by the emotional valence of the stimuli used in the studies, since not all the studies comprising the insula cluster contained emotional content, such as negative feedback. They share, however, a high level of uncertainty in their paradigms, e.g., temporal uncertainty or ambiguous stimuli or categories. The processing of uncertainty has also been shown to be associated with insula activity (Simmons et al., 2008; Sarinopoulos et al., 2010), which could be interpreted as an aversive and thus emotional state.

### Integration into a Clinical Model of Expectation Change and Persistence

Rief et al.’s (2015) model proposes that following an expectation violation, various information processing mechanisms decide whether an expectation is changed and integrated or maintained and reinforced. In order to shed light on the cognitive processes involved in an expectation change following an expectation violation, we investigated the brain areas more active in paradigms encouraging a behavioral strategic change following a prediction error.

The striatum might be involved in learning the specific contingencies between stimulus and outcome. This might eventually form an expectation about the action strategies resulting in a rewarding outcome. However, when facing an expectation violation, the caudate body might signal a non-rewarding outcome, even though the same behavioral strategy has been employed. On the other hand, if the environment encourages a passive behavior, i.e., no action has to be taken following an expectation violation, the individual is not required to determine a behavioral alternative. The difference in expectation and outcome could be solved by mechanisms such as immunization, leading to an expectation persistence (Kube et al., 2016). In contrast, if the environment encourages or even enforces the use of action alternatives, e.g., an active prediction error paradigm or a therapeutic setting, a behavioral reaction to the situation would be necessary. In such a case, the putamen could be involved in determining a novel behavior and initiate the action alternative by projecting to the medial globus pallidus. This structure could then initiate the motoric aspect of the action alternative. The thalamus, the centromedian nucleus, and the pedunculopontine nucleus could be involved in assessing the reward when employing the new behavior. If the action alternative leads to a satisfying result, i.e., a rewarding outcome, the behavior is integrated and leads to an expectation change.

The involvement of the insula especially in prediction error paradigms encouraging behavioral adaptation suggests an emotional component to be important. Due to its association with avoidance learning, the insula might be involved in assessing aversive outcomes following an action alternative. This contrasts with the reward assessment of thalamus, centromedian nucleus and pedunculopontine nucleus. It might be possible, that the avoidance of an unwanted outcome, e.g., a negative emotional state, is as important as the gain of a rewarding outcome. The aversion of a negative emotional state might be a rewarding outcome in itself which has to be considered when assessing the reward of an action alternative.

### Limitations

A few methodological limitations have to be considered. First, the studies we included used various paradigms, showing a wide range of stimuli, tasks and underlying mathematical models. However, there is evidence of different brain activity involved in various types of learning and in particular in model-based (i.e., goal-directed actions) vs. model-free (i.e., habit-based actions) approaches (Maia, 2009; Wunderlich et al., 2012). Moreover, the ALE meta-analysis itself has a few limitations. Coordinate-based analyses accumulate power across studies (Costafreda, 2009) and cannot reproduce the same quality in results as image-based meta-analyses (Salimi-Khorshidi et al., 2009). A third limitation is that the results of the subtraction analysis do not survive a FDR corrected threshold. Considering this restriction, the results of the contrast analysis have to be interpreted with caution. Eickhoff et al. (2016) recommend a minimal sample size of 17 studies for the ALE meta-analysis. We could only include 19 studies using a passive observational paradigm. This suggests that the statistical power may be rather small for the subtraction analysis, explaining why our results did not survive the FDR corrected threshold. For future research, it is necessary to repeat the analyses with a larger study sample to increase the statistical power. This will allow a more decisive analysis of the differences in neurological activity between active behavioral and passive observational prediction error paradigms.

## Conclusion

This meta-analysis sheds light into the cognitive processes involved in the execution of action alternatives following an expectation violation. The information processing involved is strongly associated with reward evaluation of newly found behavioral adaptations. However, further research is needed in order to explicitly investigate the expectations of participants of prediction error paradigms regarding their behavioral strategies.

## Author Contributions

LD: Did the major part of the work regarding conception and methodology of the article; performed the analyses and the evaluation of the results; approves of the manuscript being published; agrees on being accountable for all aspects of the work, ensuring that questions regarding quality and accuracy of the work are investigated appropriately and resolved. WR: Substantially contributed to the conception of the article, revised the manuscript critically and contributed to the content; approves of the manuscript being published; agrees on being accountable for all aspects of the work, ensuring that questions regarding quality and accuracy of the work are investigated appropriately and resolved.

## Conflict of Interest Statement

The authors declare that the research was conducted in the absence of any commercial or financial relationships that could be construed as a potential conflict of interest.

## Footnotes

1 http://www.ncbi.nlm.nih.gov/pubmed

2 http://apps.webofknowledge.com/

3 http://neurosynth.org/

4 http://ric.uthscsa.edu/mango/papaya.html

5 http://www.brainmap.org./ale/
